# Supplementary material for: OpenMM 7: Rapid development of high performance algorithms for molecular dynamics
Source: PLoS Comput Biol. 2017 Jul 26;13(7):e1005659. doi: 10.1371/journal.pcbi.1005659 (PMC5549999; doi:10.1371/journal.pcbi.1005659)
Supplement: S1 Source Code — (ZIP) [file pcbi.1005659.s002.zip › OpenMM-7.0.1-Source/docs/api-python/generated/simtk.openmm.openmm.GBSAOBCForce.html]

GBSAOBCForce — OpenMM 7.0.1.dev-5e86c4f documentation


# GBSAOBCForce¶

*class* `simtk.openmm.openmm.``GBSAOBCForce`(*\*args*)¶
:   This class implements an implicit solvation force using the GBSA-OBC model.

    To use this class, create a GBSAOBCForce object, then call addParticle() once for each particle in the System to define its parameters. The number of particles for which you define GBSA parameters must be exactly equal to the number of particles in the System, or else an exception will be thrown when you try to create a Context. After a particle has been added, you can modify its force field parameters by calling setParticleParameters(). This will have no effect on Contexts that already exist unless you call updateParametersInContext().

    When using this Force, the System should also include a NonbondedForce, and both objects must specify identical charges for all particles. Otherwise, the results will not be correct. Furthermore, if the nonbonded method is set to CutoffNonPeriodic or CutoffPeriodic, you should call setReactionFieldDielectric(1.0) on the NonbondedForce to turn off the reaction field approximation, which does not produce correct results when combined with GBSA.

    `__init__`(*self*) → GBSAOBCForce¶
    :   \_\_init\_\_(self, other) -> GBSAOBCForce

        Create a GBSAOBCForce.

    Methods

    |  |  |
    | --- | --- |
    | `__init__`((self) -> GBSAOBCForce) | \_\_init\_\_(self, other) -> GBSAOBCForce |
    | `addParticle`((self, charge, radius, ...) | Add the GBSA parameters for a particle. |
    | `getCutoffDistance`((self) -> double) | Get the cutoff distance (in nm) being used for nonbonded interactions. |
    | `getForceGroup`((self) -> int) | Get the force group this Force belongs to. |
    | `getNonbondedMethod`(...) | Get the method used for handling long range nonbonded interactions. |
    | `getNumParticles`((self) -> int) | Get the number of particles in the system. |
    | `getParticleParameters`(self, index) | Get the force field parameters for a particle. |
    | `getSoluteDielectric`((self) -> double) | Get the dielectric constant for the solute. |
    | `getSolventDielectric`((self) -> double) | Get the dielectric constant for the solvent. |
    | `getSurfaceAreaEnergy`((self) -> double) | Get the energy scale for the surface energy term, measured in kJ/mol/nm^2. |
    | `setCutoffDistance`(self, distance) | Set the cutoff distance (in nm) being used for nonbonded interactions. |
    | `setForceGroup`(self, group) | Set the force group this Force belongs to. |
    | `setNonbondedMethod`(self, method) | Set the method used for handling long range nonbonded interactions. |
    | `setParticleParameters`(self, index, charge, ...) | Set the force field parameters for a particle. |
    | `setSoluteDielectric`(self, dielectric) | Set the dielectric constant for the solute. |
    | `setSolventDielectric`(self, dielectric) | Set the dielectric constant for the solvent. |
    | `setSurfaceAreaEnergy`(self, energy) | Set the energy scale for the surface energy term, measured in kJ/mol/nm^2. |
    | `updateParametersInContext`(self, context) | Update the particle parameters in a Context to match those stored in this Force object. |
    | `usesPeriodicBoundaryConditions`((self) -> bool) | Returns whether or not this force makes use of periodic boundary conditions. |

    Attributes

    |  |  |
    | --- | --- |
    | `CutoffNonPeriodic` |  |
    | `CutoffPeriodic` |  |
    | `NoCutoff` |  |

    `getNumParticles`(*self*) → int¶
    :   Get the number of particles in the system.

    `addParticle`(*self*, *charge*, *radius*, *scalingFactor*) → int¶
    :   Add the GBSA parameters for a particle. This should be called once for each particle in the System. When it is called for the i’th time, it specifies the parameters for the i’th particle.

        |  |  |
        | --- | --- |
        | Parameters: | - **charge** (*double*) – the charge of the particle, measured in units of the proton charge - **radius** (*double*) – the GBSA radius of the particle, measured in nm - **scalingFactor** (*double*) – the OBC scaling factor for the particle |
        | Returns: | the index of the particle that was added |
        | Return type: | int |

    `getParticleParameters`(*self*, *index*)¶
    :   Get the force field parameters for a particle.

        |  |  |
        | --- | --- |
        | Parameters: | **index** (*int*) – the index of the particle for which to get parameters |
        | Returns: | - **charge** (*double*) –   the charge of the particle, measured in units of the proton charge - **radius** (*double*) –   the GBSA radius of the particle, measured in nm - **scalingFactor** (*double*) –   the OBC scaling factor for the particle |

    `setParticleParameters`(*self*, *index*, *charge*, *radius*, *scalingFactor*)¶
    :   Set the force field parameters for a particle.

        |  |  |
        | --- | --- |
        | Parameters: | - **index** (*int*) – the index of the particle for which to set parameters - **charge** (*double*) – the charge of the particle, measured in units of the proton charge - **radius** (*double*) – the GBSA radius of the particle, measured in nm - **scalingFactor** (*double*) – the OBC scaling factor for the particle |

    `getSolventDielectric`(*self*) → double¶
    :   Get the dielectric constant for the solvent.

    `setSolventDielectric`(*self*, *dielectric*)¶
    :   Set the dielectric constant for the solvent.

    `getSoluteDielectric`(*self*) → double¶
    :   Get the dielectric constant for the solute.

    `setSoluteDielectric`(*self*, *dielectric*)¶
    :   Set the dielectric constant for the solute.

    `getSurfaceAreaEnergy`(*self*) → double¶
    :   Get the energy scale for the surface energy term, measured in kJ/mol/nm^2.

    `setSurfaceAreaEnergy`(*self*, *energy*)¶
    :   Set the energy scale for the surface energy term, measured in kJ/mol/nm^2.

    `getNonbondedMethod`(*self*) → OpenMM::GBSAOBCForce::NonbondedMethod¶
    :   Get the method used for handling long range nonbonded interactions.

    `setNonbondedMethod`(*self*, *method*)¶
    :   Set the method used for handling long range nonbonded interactions.

    `getCutoffDistance`(*self*) → double¶
    :   Get the cutoff distance (in nm) being used for nonbonded interactions. If the NonbondedMethod in use is NoCutoff, this value will have no effect.

        |  |  |
        | --- | --- |
        | Returns: | the cutoff distance, measured in nm |
        | Return type: | double |

    `setCutoffDistance`(*self*, *distance*)¶
    :   Set the cutoff distance (in nm) being used for nonbonded interactions. If the NonbondedMethod in use is NoCutoff, this value will have no effect.

        |  |  |
        | --- | --- |
        | Parameters: | **distance** (*double*) – the cutoff distance, measured in nm |

    `updateParametersInContext`(*self*, *context*)¶
    :   Update the particle parameters in a Context to match those stored in this Force object. This method provides an efficient method to update certain parameters in an existing Context without needing to reinitialize it. Simply call setParticleParameters() to modify this object’s parameters, then call updateParametersInContext() to copy them over to the Context.

        The only information this method updates is the values of per-particle parameters. All other aspects of the Force (the nonbonded method, the cutoff distance, etc.) are unaffected and can only be changed by reinitializing the Context. Furthermore, this method cannot be used to add new particles, only to change the parameters of existing ones.

    `usesPeriodicBoundaryConditions`(*self*) → bool¶
    :   Returns whether or not this force makes use of periodic boundary conditions.

        |  |  |
        | --- | --- |
        | Returns: | true if force uses PBC and false otherwise |
        | Return type: | bool |

    `__delattr__`¶
    :   x.\_\_delattr\_\_(‘name’) <==> del x.name

    `__format__`()¶
    :   default object formatter

    `__getattribute__`¶
    :   x.\_\_getattribute\_\_(‘name’) <==> x.name

    `__hash__`¶

    `__reduce__`()¶
    :   helper for pickle

    `__reduce_ex__`()¶
    :   helper for pickle

    `__sizeof__`() → int¶
    :   size of object in memory, in bytes

    `__str__`¶

    `getForceGroup`(*self*) → int¶
    :   Get the force group this Force belongs to.

    `setForceGroup`(*self*, *group*)¶
    :   Set the force group this Force belongs to.

        |  |  |
        | --- | --- |
        | Parameters: | **group** (*int*) – the group index. Legal values are between 0 and 31 (inclusive). |

High performance molecular simulation on GPUs


### Navigation

- Application Layer
- Library Layer
  - Core Objects
  - Forces
  - Integrators
  - Extras

©2015, Stanford University and the Authors.
|
Powered by Sphinx 1.3.1
& Alabaster 0.7.7
|
Page source
